# Redescription of *Borboropactusjiangyong* Yin, Peng, Yan & Kim, 2004 (Araneae, Thomisidae), with the first description of the male

**DOI:** 10.3897/zookeys.870.35230

**Published:** 2019-08-07

**Authors:** Ze-Yuan Meng, Hui-Pu Luo, Yong-Hong Xiao, Xiang Xu, Ke-Ke Liu

**Affiliations:** 1 College of Life Science, Jinggangshan University, Ji’an 343009, Jiangxi, China; 2 College of Life Science, Hunan Normal University, Changsha 410081, Hunan, China; 3 The National & Local Joint Engineering Laboratory of Animal Peptide Drug Development (Hunan Normal University), National Development and Reform Commission, Changsha, Hunan 410081, China

**Keywords:** China, digging spiders, distribution, Jiangxi Province, Jinggang Mountain, taxonomy

## Abstract

The male of *Borboropactusjiangyong* Yin, Peng, Yan & Kim, 2004 is described for the first time from Jinggang Mountain, Ji’an City, Jiangxi Province, China. Based on male and female specimens, the species is redescribed, comprehensively illustrated, and its geographic distribution in China is delimited and discussed.

## Introduction

Species in the genus *Borboropactus* Simon, 1884 are digging spiders, usually living in leaf litter, woody debris, tree bark, or on the forest ground, in a wide range of habitats, including tropical forests, subtropical forests, temperate forests, and early Tertiary Baltic amber (Wunderlich 2004a, b; Benjamin et al. 2008; Tang and Li 2010; Benjamin 2011; Yin et al. 2012; Ramírez 2014; World Spider Catalog 2019). Species can be easily recognised by a combination of somatic characters (Wunderlich 2004a, b; Benjamin et al. 2008; Benjamin 2011; Ramírez 2014), namely their body with adhered soil particles, numerous club-shaped hairs and powerful and long leg I.

The genus was established based on the female specimens of *B.squalidus* Simon, 1884 collected from West Africa (Simon 1884). Subsequently, it was transferred in the genus *Regillus* O. Pickard-Cambridge, 1884 by Simon in 1895, along with another two species, namely *B.bituberculatus* Simon, 1884 and *Thomisusvulcanicus* Doleschall, 1859. Wunderlich (2004a, b) elevated *Borboropactus* to family rank, presumably due to its presence in Baltic amber and its unique? digging behavior. The new family Borboropactidae was subsequently rejected by Benjamin et al. (2008) based on the results of molecular phylogenetic analysis of three concatenated gene sequences (mtDNA 16S rRNA and cytochrome *c* oxidase subunit I and nuc DNA histone 3) of 25 genera of crab spiders and eleven out-groups. The molecular results were confirmed by a cladistic analysis of morphological data (Benjamin 2011). Based on molecular, morphological and additional ultrastructural characters, the genus *Borboropactus* is currently considered a specific lineage within Thomisidae (Benjamin et al. 2008; Benjamin 2011; Ramírez 2014). However, an enormous amount of work recently also shows that *Borboropactus* species are weakly supported among 44 terminals based on mitochondrial (12S, 16S, COI) and nuclear (histone H3, 18S, 28S) genomes, a result compatible with the family level recognition proposal of Wunderlich (2004b) (Wheeler et al. 2017).

Currently, there are 17 nominal species within *Borboropactus* (World Spider Catalog 2019), inhabiting tropical Africa and Asia. Taxonomic species identification is challenging because most species are described based either on single females or males, including *B.asper* (O. Pickard-Cambridge, 1884) (female), *B.australis* (Lawrence, 1937) (female), *B.biprocessus* Tang, Yin & Peng, 2012 (male), *B.cinerascenssumatrae* (Strand, 1907) (female), *B.elephantus* (Tikader, 1966) (female), *B.javanicola* (Strand, 1913) (female), *B.jiangyong* Yin, Peng, Yan & Kim, 2004 (female), *B.longidens* Tang & Li, 2010 (female), *B.silvicola* (Lawrence, 1938) (female), *B.squalidus* Simon, 1884 (female), and *B.vulcanicus* (Doleschall, 1859) (female). The genus is represented by six species in China (World Spider Catalog 2019), half of which are also only known from either the male or female.

After examining spider specimens collected using the sieving method from the Jinggang Mountain National Nature Reserve in the past six years, the presumed male of *B.jiangyong* was found, and is here described for the first time. Additionally, female specimens belonging to this species have been identified among material collected from Yunnan, Guangdong and Hunan. The newly studied material allows for a more precise delimitation of the distribution of *B.jiangyong*. This study further includes photographs, SEM illustrations, and line drawings to provided more complete and detailed information of the somatic and genital morphology of this interesting species.

## Materials and methods

Specimens were examined using a Zeiss Stereo Discovery V12 stereomicroscope with Zoom Microscope System. Further details were studied using a Zeiss Axio Scope A1 compound microscope with a KUY NICE CCD. Both the male palps and female genitalia were detached from the spider body and observed in 80−85% ethanol. For SEM photographs, the specimens were kept under natural dry conditions and photographed with a ZEISS EVO LS15 scanning electron microscope. The specimens were stored in 80% ethanol after SEM.

All morphological measurements were taken using a stereomicroscope (AxioVision SE64 Rel. 4.8.3) and given in millimetres. The body length of each specimen does not include the spinnerets. Leg measurements are given as total length (femur, patella, tibia, metatarsus, tarsus).

Terminology of the male and female genitalia follows Wunderlich (2004a, b), Benjamin et al. (2008), Benjamin (2011), and Ramírez (2014). Leg spines were documented by dividing each leg segment into two aspects, dorsal and ventral, the latter being divided into prolateral and retrolateral, e.g., I femur 0 (dorsal) 2 (prolateral ventral) 2 (retrolateral ventral); I tibia 1 (dorsal) 4 (prolateral ventral) 4 (retrolateral ventral). The abbreviations used in the text and figures are as follows:

**Eyes**

**ALE** anterior lateral eye;

**AME** anterior median eye;

**MOA** median ocular area

**PLE** posterior lateral eye;

**PME** posterior median eye;

**Male palp**

**C** conductor;

**E** embolus;

**RTA** retrolateral tibial apophysis;

**MA** median apophysis;

**CD** copulatory duct;

**CO** copulatory opening;

**Epygine**

**ET** epigynal teeth;

**LL** epigynal lateral lobe;

**MF** median field;

**S** spermathecae;

**Legs**

**fe** femur;

**me** metatarsus;

**pa** patella;

**ta** tarsus;

**ti** tibia

## Taxonomy

### Family Thomisidae Sundevall, 1833

#### Genus Borboropactus

Taxon classificationAnimaliaAraneaeThomisidae

Simon, 1884

247071EEC47058E98D248FD06362D31F

##### Diagnosis.

This genus can be easily distinguished from other genera in thomisid spiders by the body covered with numerous club-shaped hairs; posterior eye row recurved in dorsal view; chelicerae toothed on both margins and with small teeth within its furrow; leg I powerful and distinctly the longest with the femur thickened prolaterally, bearing tubercles/spines, and with a depression; legs I and II with paired ventral tibial and metatarsal spines; legs III and IV without spines; metatarsal trichobothria with expansion, tarsal on sensory field with bumps in a long unexpanded area; presence of the tarsal pit organ (a special large leg sense organ); proclaw with a special patch of teeth; female epigynum teeth well developed; male palpal tibia with a retrodistal apophysis, bulbus simple and prominent, median apophysis present, conductor present or absent. See also Wunderlich (2004b) for a diagnosis of the genus and Wunderlich (2004a), Benjamin et al. (2008), Benjamin (2011), Ramírez (2014) and this paper for species descriptions.

#### Borboropactus jiangyong

Taxon classificationAnimaliaAraneaeThomisidae

Yin, Peng, Yan & Kim, 2004

2208BD4ADF2B5E69849ACE743473B46E

**Figure 1.**
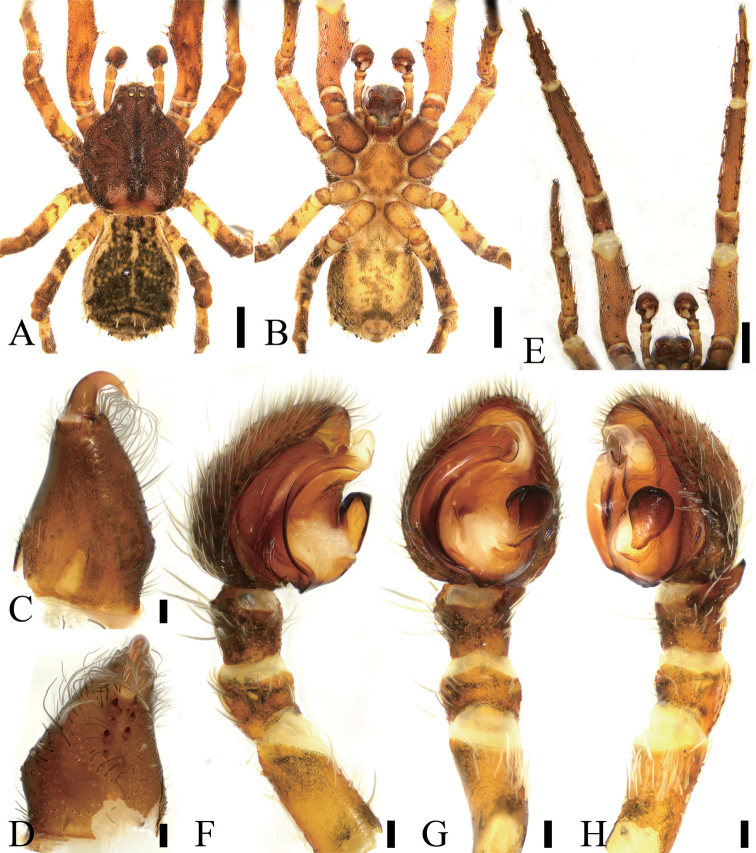
*Borboropactusjiangyong* Yin, Peng, Yan & Kim, 2004, male. **A** Habitus, dorsal view **B** habitus, ventral view **C** left chelicera, dorsolateral view **D** same, ventral view **E** left leg I and II, ventral view **F** left palp, prolateral view **G** left palp, ventral view **H** left palp, retrolateral view. Scale bars: 1 mm (**A, B, E**), 0.1 mm (**C, D, F–H**).

**Figure 2.**
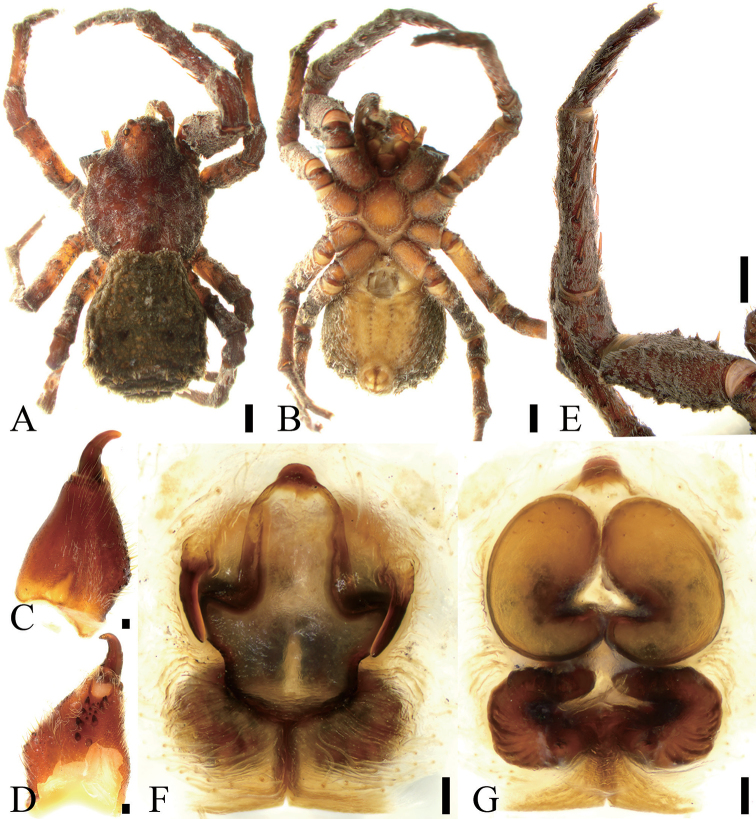
*Borboropactusjiangyong* Yin, Peng, Yan & Kim, 2004, female. **A** Habitus, dorsal view **B** habitus, ventral view **C** left chelicera, dorsolateral view **D** same, ventral view **E** left leg I, prolateral view **F** epigyne, ventral view **G** vulva, dorsal view. Scale bar: 1 mm (**A, B, E**), 0.1 mm (**C, D, F, G**).

**Figure 3.**
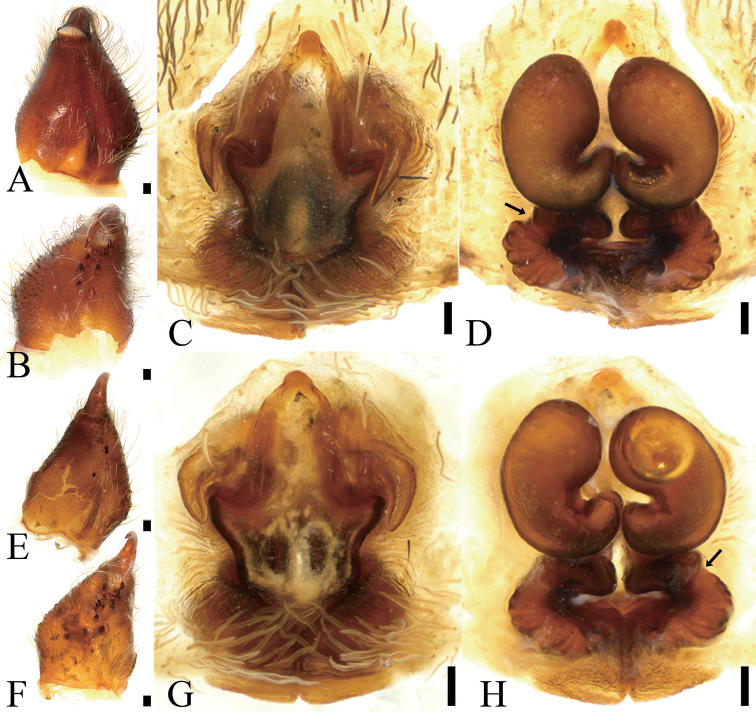
*Borboropactusjiangyong* Yin, Peng, Yan & Kim, 2004, female chelicera and genitalia. **A** Left chelicera, dorsolateral view **B** same, ventral view **C** epigyne, ventral view **D** vulva, dorsal view, black arrow shows anterior part of spermathecae with a constriction **E** left chelicera, dorsolateral view **F** same, ventral view **G** epigyne, ventral view **H** vulva, dorsal view; black arrow shows anterior part of spermathecae with a constriction. Scale bars: 0.1 mm (**A–H**).

**Figure 4.**
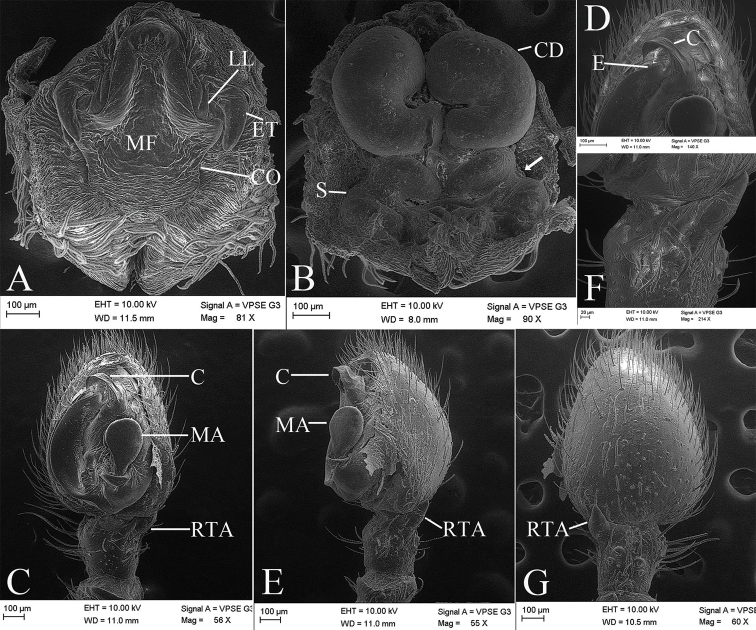
*Borboropactusjiangyong* Yin, Peng, Yan & Kim, 2004, SEMs of female and male genitalia. **A** Epigyne, ventral view **B** vulva, dorsal view, black arrow shows anterior part of spermathecae with a constriction **C** left palp, ventral view **D** detail of embolus and conductor **E** left palp, retrolateral view **F** detail of RTA, retrolateral view **G** left palp, dorsal view. Abbreviations: C – conductor, CD – copulatory duct, CO – copulatory opening, E – embolus, ET – epigynal teeth, LL – epigynal lateral lobe, MA – median apophysis, MF – median field, RTA – retrolateral tibial apophysis, S – spermathecae.

**Figure 5.**
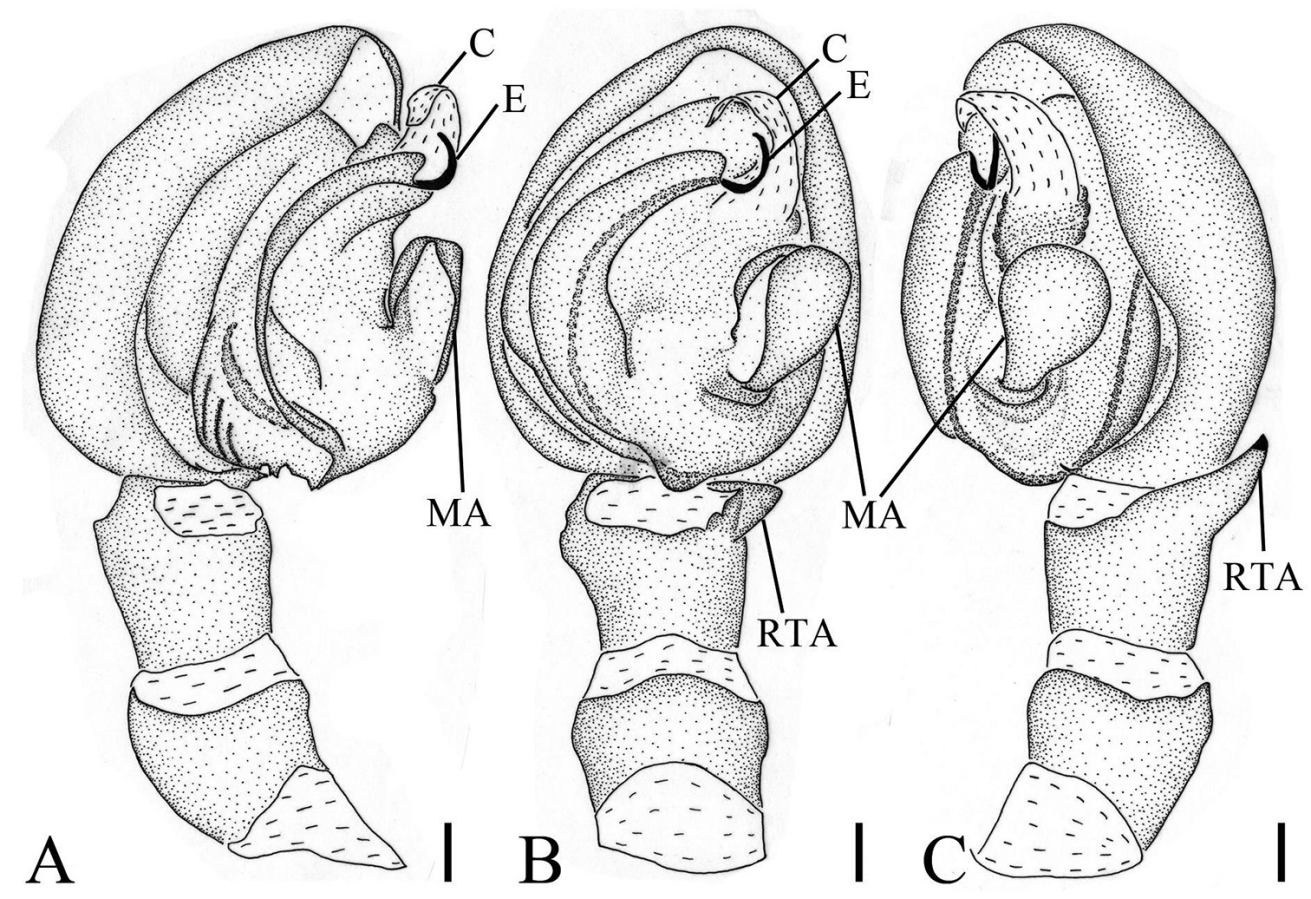
*Borboropactusjiangyong* Yin, Peng, Yan & Kim, 2004, male palp. **A** Left palp, prolateral view **B** left palp, ventral view **C** left palp, retrolateral view. Scale bars: 0.1 mm (**A–C**). Abbreviations: C – conductor, E – embolus, MA – median apophysis, RTA – retrolateral tibial apophysis.

**Figure 6.**
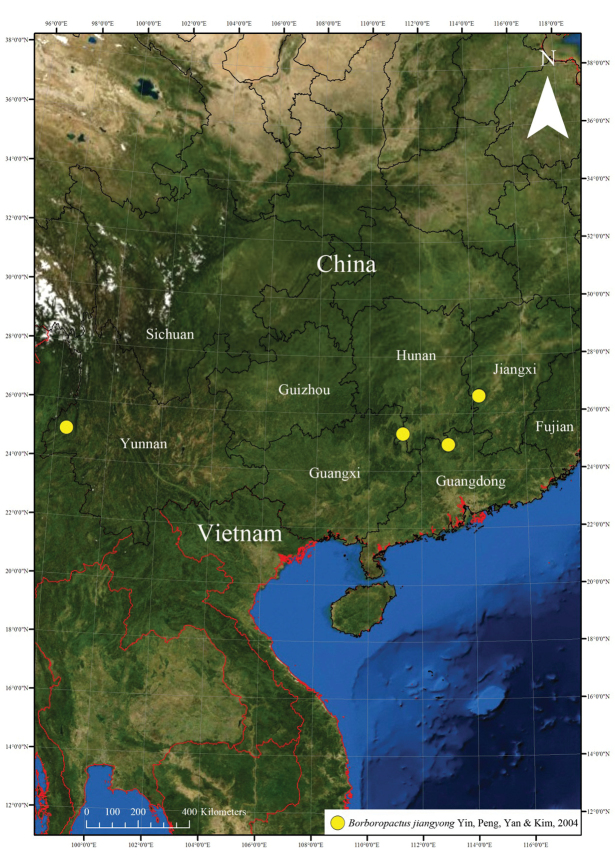
Collection localities of *Borboropactusjiangyong* Yin, Peng, Yan & Kim, 2004 in China.

Figs 1 , 2 , 3 , 4 , 5 , 6

Borboropactus jiangyong Yin et al., 2004b: 27, figs 1–5; Yin et al., 2012: 1259, fig. 676a–e. Holotype not examined (see below in Remarks)

##### Material examined.

1 ♀, 1 ♂ (JGSU), China: *Jiangxi Province*, Ji’an City, Jinggangshan County Level City, Luofu Town, Xiangzhou Village, Jinggang Mountain National Nature Reserve, 26°36'10.31"N, 114°15'15.52"E, 360 m, leaf litter, 5.X.2018, Ke-ke Liu and Hui-pu Luo leg.; as previous, 1 ♀ (JGSU), 26°36'10.8"N, 114°15'28.8"E, 375 m, leaf litter, 5 July 2017, Ke-ke Liu, Zhi-wu Chen, Ze-yuan Meng and Wen-jun Xie leg.; as previous, 1 ♀ (JGSU), 26°37'19.20"N, 114°15'54"E, 460 m, leaf litter, 6 August 2015, Ke-ke Liu, Sha Wu, Ze-yuan Meng, Ce Xu and Shi-cong He leg.; 1 ♀ (HNU), *Yunnan Province*, Baoshan City, Tengchong Town, Shangying Village, Chuanlong, 25°0'8.65"N, 098°25'24.85"E, 2000 m, 4 June 2006, Chang-min Yin, Jia-fang Hu and Xiao-hua Yang leg.; 1 ♀ (HNU), *Guangdong Province*, Shaoguan City, Ruyuan Town, Nanling National Nature Reserve, Qinshuigu, 24°55'07.89"N, 113°02'30.58"E, 830 m, 25 July 2016, Hai-qiang Yin, Tie-yang Zhou, Gu-chun Zhou, Chao-min Li, Ai-lan He, Wang Liu, Jin-xin Liu, Zhuo-er Chen and Chen Zeng leg.

##### Diagnosis.

The male of this species resembles both *B.biprocessus* Tang, Yin & Peng, 2012 and *B.bituberculatus* by the ear-shaped median apophysis on the tegulum, but can be separated by cheliceral teeth number, 4 promarginal and 3 retromarginal, instead of 4 and 4 in species *B.biprocessus* and 4 and 5 in species *B.bituberculatus*; a strong RTA extending dorsally on palp cymbium, instead of relative thinner in species *B.biprocessus* and stouter in species *B.bituberculatus*, and the relatively narrowed membranous conductor, which is broad in species *B.biprocessus* and *B.bituberculatus* (Figs 1C, D, F–H, 4C–G, 5) (see also Yin et al. 2012: 1258, fig. 675A–C; Tang and Li 2010: 18–20, figs 12A–D, 14A, B). Females are separated from similar species of *B.bituberculatus* Simon, 1884 and *B.brevidens* Tang & Li, 2010 by the shape of the broad, cruciform median field (tongue-shaped in *B.bituberculatus*; triangular in *B.brevidens*), chelicerae with four promarginal teeth and 4 or 3 retromarginal teeth (4 pro- and 5 retromarginal teeth in *B.bituberculatus*; 5 pro- and 4 retromarginal teeth in *B.brevidens*) , longer epigynal teeth (relative shorter in *B.bituberculatus* and *B.brevidens*), and the enlarged, strongly twisted copulatory duct (Figs 2C, D, F, G, 3, 4A, B) (see also Tang and Li 2010: 8–20, figs 3A–C, 6C, D, 13A–D, 14C, D).

##### Description.

***Male.****Habitus* as in Fig. 1A, B. Total length 6.64. Prosoma (Fig. 1A) length 3.31, width 2.71. Eye (Fig. 1A) sizes and interdistances: AME 0.17; ALE 0.17; PME 0.17; PLE 0.20; AME–AME 0.08; AME–ALE 0.10; PME–PME 0.11; ALE–ALE0.54; PME–PLE 0.19; PLE–PLE 0.73; ALE−PLE 0.10, AME−PME 0.08; AME–PLE 0.33. MOA: 0.39 long; 0.41 front width, 0.46 back width, anterior and posterior eye row recurved, anteriorly located on prosoma. Chelicerae (Fig. 1C, D) with four promarginal teeth and four retromarginal teeth and including a vestige tooth, and two small denticles in-between teeth. Endites (Fig. 1B) nearly quadrilateral. Labium (Fig. 1B) rectangular, anteriorly with 6–10 strong setae. Sternum (Fig. 1B) shield-shaped, with abundant setae around margin. Leg (Fig. 1A, B, E) measurements: I 11.33 (3.50, 1.19, 3.61, 2.06, 0.97); II 7.88 (2.19, 0.93, 2.24, 1.59, 0.93); III 7.27 (2.18, 0.78, 1.90, 1.60, 0.81); IV 8.93 (2.86, 0.97, 2.14, 1.87, 1.09). Leg formula 1423. Spination: I fe 120, pa 001, ti 055, met 033, ta 000; II fe 000, pa 000, ti 044, met 033, ta 000. Fe I with six ventral cusps. Opisthosoma length 3.17, width 2.37, hardened, with abundant particles and two pairs of rugose sigillae, posteriorly with many clavate hairs on dorsal view.

*Colouration and pattern.* Prosoma pyriform, yellow brown, densely covered white feathery setae, with a longitudinal dark stripe, clustered short hairs at back of the PLE and near the posterior of the stripe, and four paired radial striae around fovea. Chelicerae, endites, and labium yellow brown. Sternum from yellow brown to orange. Legs from yellow to orange. Opisthosoma from orange to greyish black, with light longitudinal stripe and abundant dark and light spots.

*Palp* (Figs 1F–H, 4C–G, 5). Palp with a relative long and strong RTA, extending dorsally; cymbium laterally protruded near the RTA; tegulum with an ear-shaped median apophysis; embolus twisted, apex slender; conductor translucent with wrinkled base.

***Female.****Habitus* as in Fig. 2A, B. Total length 13.35. Prosoma (Fig. 2A) length 6.64, width 5.87. Eye (Fig. 2A) sizes and interdistances: AME 0.22; ALE 0.24; PME 0.24; PLE 0.26; AME–AME 0.25; AME–ALE 0.33; PME–PME 0.23; ALE–ALE 1.28; PME–PLE 0.60; PLE–PLE 1.79; ALE−PLE 0.26, AME−PME 0.29; AME–PLE 0.75. MOA: 0.67 long; 0.62 front width, 0.69 back width. Chelicerae (Fig. 2C, D) with four promarginal teeth and three retromarginal teeth and including a vestige tooth, and six small denticles in-between teeth. Endites (Fig. 2B) nearly quadrilateral. Labium (Fig. 2B) rectangular, anteriorly with 8 strong setae. Sternum (Fig. 2B) shield-shaped, with abundant setae around margin. Leg (Fig. 2A, B, E) measurements: I 16.68 (5.64, 2.51, 4.68, 2.58, 1.27); II 11.54 (3.77, 1.69, 3.02, 2.04, 1.02); III 11.40 (3.29, 1.66, 3.24, 2.30, 0.91); IV 12.26 (4.22, 1.53, 3.07, 2.45, 0.99). Leg formula 1423. Spination: I fe 220, pa 000, ti 055, met 033, ta 000; II fe 000, pa 000, ti 044, met 033, ta 000. Fe I with fourteen ventral cusps. Opisthosoma length 6.71, width 5.87.

*Colouration and pattern*. Prosoma orange or red-brown, without stripe. Opithosoma without clear stripe.

*Epigynum* (Fig. 2F, G). Median field cruciform, broad, delimited by furrows; epigynal lateral lobe long, arising from antero-lateral part of median field; epigynal teeth well advanced, situated anteriorly, arising bilaterally, sharp, slightly shorter than 1/2 median field length; copulatory ducts broad, wider than spermathecae, both ends swollen, C-shaped, located at anterior of vulva, anterior and posterior part are approaching each other; spermathecae curved laterally, tube-shaped, anterior part not have a constriction, median part C-shaped, both ends slightly swollen, a pair of wrinkled chitinous lamellae covered the posterior part of spermathecae.

##### Distribution.

Known from Hunan, Yunnan, Jiangxi, and Guangdong provinces (Fig. 6). Specimens were collected from an area approaching 26°N in China (Fig. 6). It is possible that this species is also distributed in Guizhou, Fujian, and Sichuan provinces. This will have to be confirmed by future collecting and further research.

##### Variability.

We conducted a survey of *Borboropactusjiangyong* female specimens from Hunan, Yunnan, Jiangxi, and Guangdong provinces in China available in museums. The detailed study of these specimens (Figs 2F, G, 3C, D, G, H, 4A, B) revealed that they all differ in the number of cheliceral denticles depending upon locality.Specimens from Yunnan bore four small denticles close to promarginal teeth and two close to retromarginal teeth; specimens from Jiangxi bore four small denticles close to promarginal teeth and four close to retromarginal teeth; or one small denticle close to promarginal teeth, three close to retromarginal teeth; or one small denticle close to promarginal teeth, four close to retromarginal teeth; from Guangdong one small denticle near promarginal teeth, one near retromarginal teeth. Similarly, specimens from different provinces also differed in the number of ventral cusps on their fe I: fourteen from Yunnan; twelve from Jiangxi; twenty-three from Jiangxi; fourteen from Jiangxi; and ten from Guangdong. Meanwhile, their body sizes, eye sizes and eye interdistances are also in the range such as total length 9.48–14.37; prosoma length 5.50–6.65, width 4.74–5.87; opisthosoma length 3.98–8.04, width 3.48–5.87; AME 0.16–0.22; ALE 0.17–0.24; PME 0.17–0.24; PLE 0.17–0.26; AME–AME 0.24–0.33; AME–ALE 0.24–0.33; PME–PME 0.20–0.30; ALE–ALE 1.01–1.38; PME–PLE 0.46–0.60; PLE–PLE 1.56–1.83; ALE−PLE 0.22–0.39, AME−PME 0.22–0.45; AME–PLE 0.59–0.86; MOA 0.59–0.76 long, 0.53–0.65 front width, 0.64–0.71 back width; leg I 14.77–18.12; II 10.20–12.30; III 10.22–11.99; IV 11.23–13.28. Finally, variability was also observed in the epigynal teeth, which may either have a sharp tip or a blunt tip; and the anterior part of spermathecae, which may or may not have a constriction. The regional variability observed across the distribution of *Borboropactusjiangyong* may be the result of the influence of environmental factors, such as temperature, elevation or habitat.

##### Remarks.

Unfortunately, the holotype of this species stored at the College of Life Sciences, Hunan Normal University (HNU), could not be studied because it was destroyed by slime moulds. However, the female holotype had been examined by Dr Guo Tang who contributed with many papers on crab spider taxonomy in China. Based on a comparative morphology analysis, he suggested that the female from Baoshan City in Yunnan Province was conspecific with the female holotype described in Tang’s PhD dissertation in 2008.

## Supplementary Material

XML Treatment for Borboropactus

XML Treatment for Borboropactus jiangyong
